# Identification of a novel TGF-β-miR-122-fibronectin 1/serum response factor signaling cascade and its implication in hepatic fibrogenesis

**DOI:** 10.18632/oncotarget.3652

**Published:** 2015-03-26

**Authors:** Chunxian Zeng, Yun-Long Wang, Chen Xie, Ye Sang, Tuan-Jie Li, Min Zhang, Ruizhi Wang, Qi Zhang, Limin Zheng, Shi-Mei Zhuang

**Affiliations:** ^1^ Key Laboratory of Gene Engineering of The Ministry of Education, State Key Laboratory of Biocontrol, Collaborative Innovation Center for Cancer Medicine, School of Life Sciences, Sun Yat-sen University, Guangzhou, P.R. China; ^2^ Key Laboratory of Liver Disease of Guangdong Province, The Third Affiliated Hospital, Sun Yat-sen University, Guangzhou, P.R. China

**Keywords:** hepatic fibrosis, noncoding RNA, microRNA, miR-122, fibronectin 1

## Abstract

Transforming growth factor-β (TGF-β) is a potent cytokine that promotes the development of fibrogenic cells, stimulates the expression of fibrosis-related genes, and consequently results in hepatic fibrogenesis. The involvement of miRNAs in this process remains largely unknown. We showed that miR-122 was substantially expressed in hepatic stellate cells (HSCs) and fibroblasts, the major sources of fibrogenic cells in liver tissues. Notably, exposure to TGF-β led to significant downregulation of miR-122. Furthermore, reintroduction of miR-122 suppressed TGF-β-induced expression of fibrosis-related genes, including alpha smooth muscle actin (α-SMA), fibronectin 1 (FN1) and α1 type I collagen (COL1A1), in HSCs and fibroblasts. Subsequent mechanism investigations revealed that miR-122 directly inhibited FN1 expression by binding to its 3′-untranslated region and indirectly reduced the transcription of α-SMA and COL1A1 by inhibiting the expression of serum response factor (SRF), a key transcription factor that mediated the activation of fibrogenic cells. Further *in vivo* studies disclosed that intravenous injection of miR-122-expressing lentivirus successfully increased miR-122 level and reduced the amount of collagen fibrils, FN1 and SRF in the livers of CCl_4_-treated mice. These findings disclose a novel TGF-β-miR-122-FN1/SRF signaling cascade and its implication in hepatic fibrogenesis, and suggest miR-122 as a promising molecular target for anti-fibrosis therapy.

## INTRODUCTION

Hepatic fibrosis is an outcome of the pathological response to chronic liver injuries and is characterized by the increased deposition and altered composition of extracellular matrix (ECM) [1]. In normal liver, ECM is mainly composed of collagens IV and VI, heparan sulfate proteoglycan and laminin, which provides functional and structural integrity for liver parenchyma. During hepatic fibrosis, ECM is changed from a type IV collagen-rich composition to a type I collagen- and fibronectin 1 (FN1)-rich composition, which distorts the architecture of the liver, and leads to hepatic cirrhosis and consequently hepatocellular cancer (HCC) [1].

The activation of fibrogenic cells is critical for hepatic fibrosis. Hepatic stellate cells (HSCs) and fibroblasts are considered as the primary source of fibrogenic cells in the liver [1, 2]. In response to chronic liver injuries, the expression and secretion of transforming growth factor-β (TGF-β) significantly increases. TGF-β is the most potent fibrogenic cytokine that activates the HSCs and fibroblasts to express fibrosis-related genes, including alpha smooth muscle actin (α-SMA), FN1 and α1 type I collagen (COL1A1), and consequently promotes hepatic fibrogenesis [1, 2]. Collagen type I is fibril-forming collagen and FN1 is essential for collagen fibril assembly [1, 3]. α-SMA, besides as a key molecular marker for the activated fibrogenic cells, also promotes contractile force and ECM stiffness [1, 2]. Although great efforts have been dedicated to unravel the molecular mechanisms underlying liver fibrogenesis, whether microRNAs (miRNAs) may regulate liver fibrogenesis is still poorly understood.

miR-122 is a liver-abundant miRNA and is implicated in different physiological and pathological processes in the liver, including hepatitis C virus replication [4], lipid metabolism [5], and HCC development [6-10]. However, little is known about the role of miR-122 in hepatic fibrosis, the precancerous lesion of HCC. In addition to hepatocytes, miR-122 has been detected in human skin fibroblasts, where it regulates p53 expression and cellular senescence [11, 12], suggesting that miR-122 is more widely distributed than originally thought and the biological function of miR-122 is not only restricted to hepatocytes. To date, it remains unknown whether miR-122 is deregulated during TGF-β-induced activation of fibrogenic cells and whether miR-122 can abrogate hepatic fibrogenesis.

Here, we showed that miR-122 expression was markedly reduced in the TGF-β-activated HSCs. Both *in vitro* and *in vivo* studies disclosed that miR-122 significantly suppressed the activation of fibrogenic cells and the TGF-β-induced expression of fibrosis-related genes, thus inhibiting the hepatic fibrogenesis. Our findings identify a novel TGF-β-miR-122-fibronectin 1/serum response factor signaling cascade and suggest miR-122 as a critical molecule in preventing hepatic fibrogenesis.

## RESULTS

### miR-122 inhibits TGF-β-induced expression of fibrosis-related genes

**Figure 1:**
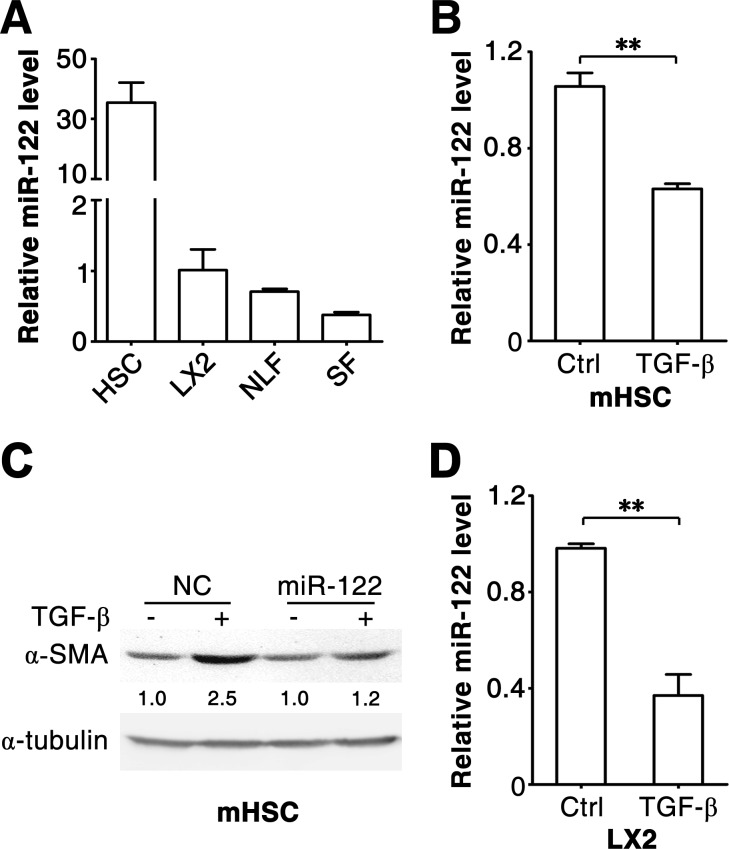
miR-122 decreases in the TGF-β-stimulated HSCs (A) The expression of miR-122 is detected in different types of cells. miR-122 expression was analyzed in mouse primary HSCs, human NLFs, SFs and LX2 cells. (B) miR-122 level decreased in TGF-β-activated primary HSCs. Mouse primary HSCs were cultured for 3 days, then exposed to 2 ng/ml TGF-β for 48 hours. (C) Restoration of miR-122 expression attenuated the TGF-β-induced expression of α-SMA in primary HSCs. Mouse primary HSCs were cultured for 3 days, then transfected with negative control (NC) or miR-122 duplex for 24 hours, followed by stimulation with 2 ng/ml TGF-β (+) or remained untreated (−) for 48 hours before immunoblotting. The intensity of each band was densitometrically quantified. The α-SMA level in each sample was normalized by that of α-tubulin (internal control). (D) miR-122 level decreased in TGF-β-stimulated LX2 cells. LX2 cells were exposed to 2 ng/ml TGF-β for 48 hours. For (A, B and D), the level of miR-122 was examined by qPCR and normalized to that of U6. ** *P* < .01.

To investigate whether miR-122 regulates TGF-β-induced activation of fibrogenic cells, we first examined its expression level in HSCs and fibroblasts, the major sources of fibrogenic cells in liver tissues. As shown, miR-122 was substantially expressed in mouse primary HSCs (Figure 1A; Supplementary Figure 1), human primary fibroblasts obtained from normal livers (NLFs) or foreskins (SFs), and an immortalized human HSC cell line, LX2 cells (Figure 1A). Notably, the level of miR-122 significantly decreased when primary HSCs were activated by TGF-β treatment (Figure 1B). Furthermore, the expression of α-SMA, a marker for fibrogenic cell activation, was upregulated in TGF-β-treated primary HSCs (Figure 1C, lanes 1 and 2), but this effect was significantly inhibited by restoration of miR-122 expression (Figure 1C). These results suggest that miR-122 downregulation may facilitate TGF-β-induced activation of HSCs.

**Figure 2:**
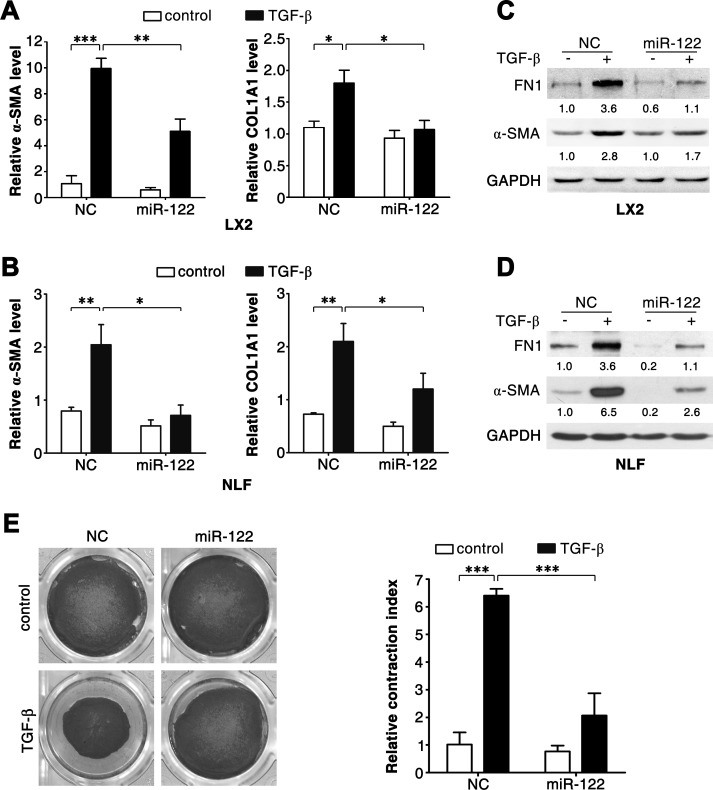
miR-122 inhibits the TGF-β-induced expression of fibrosis-related genes (A-D) Introduction of miR-122 repressed the TGF-β-stimulated expression of α*-SMA*, *COL1A1* and *FN1*. LX2 cells (A, C) and NLFs (B, D) were transfected with negative control (NC) or miR-122 duplex for 24 hours, and then stimulated with 2 ng/ml TGF-β (+) or remained untreated (control, -) for 48 hours before qPCR analysis (A, B) or immunoblotting (C, D). For qPCR analysis, the levels of target genes were normalized to the expression of *GAPDH*. For immunoblotting, the intensity of each band was densitometrically quantified. The levels of target genes in each sample were normalized by that of GAPDH (internal control). (E) Ectopic expression of miR-122 inhibited the TGF-β-promoted contraction of collagen matrix containing LX2 cells. LX2 cells transfected with the indicated duplex were embedded in collagen matrix for 1 hour, and then incubated with 2 ng/ml TGF-β or remained untreated (control) for 24 hours before collagen lattice release. The contraction index denoted the change of collagen gel size at 24 hours after lattice release. * *P* < .05; ** *P* < .01; *** *P* < .001.

Further investigations were conducted using LX2 and NLFs. Consistent with primary HSCs, TGF-β stimulation induced downregulation of miR-122 in LX2 cells (Figure 1D). As expected, TGF-β treatment resulted in increased mRNA levels of fibrosis-related genes, like α-SMA, COL1A1 and FN1, in LX2 and NLFs (Figure 2A and 2B; Supplementary Figure 2A and 2B). Interestingly, introduction of miR-122 attenuated TGF-β-induced elevation in α-SMA and COL1A1 mRNA levels (Figure 2A and 2B), but did not affect TGF-β-promoted increase of FN1 mRNA (Supplementary Figure 2A and 2B). However, ectopic expression of miR-122 abrogated TGF-β-induced upregulation of FN1 protein level in LX2 and NLFs (Figure 2C and 2D). These findings were also reproducible in SFs (Supplementary Figure 3A-D).

It is known that α-SMA promotes fibrogenic cell contraction and consequently increases ECM stiffness. We found that miR-122 attenuated TGF-β-promoted α-SMA expression at both mRNA (Figure 2A and 2B; Supplementary Figure 3A) and protein levels in LX2, NLFs and SFs (Figure 2C and 2D; Supplementary Figure 3D). Consistently, TGF-β significantly induced contraction of collagen matrix that contained LX2 cells, and this effect was significantly attenuated when LX2 cells were transfected with miR-122 duplex (Figure 2E).

Collectively, these data suggest that miR-122 may suppress TGF-β-induced activation of fibrogenic cells and in turn attenuate the expression of fibrosis-related genes.

### miR-122 directly suppresses FN1 expression and indirectly attenuates the transcription of α-SMA and COL1A1

**Figure 3:**
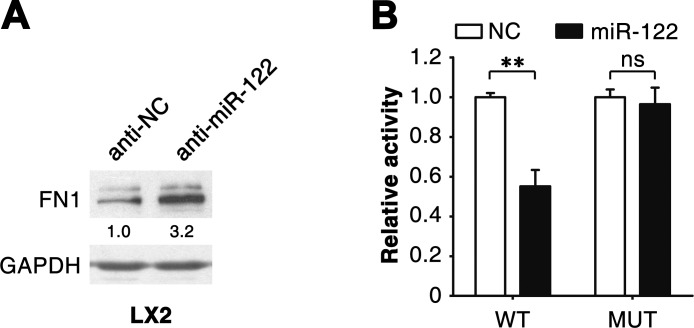
miR-122 suppresses FN1 expression by binding to its 3′UTR (A) Knockdown of endogenous miR-122 enhanced FN1 protein levels. LX2 cells were transfected with the inhibitor of miR-122 (anti-miR-122) or its negative control (anti-NC) for 48 hours before immunoblotting. The intensity of each band was densitometrically quantified. The FN1 level was normalized by that of GAPDH (internal control). (B) Expression of miR-122 inhibited the activity of the luciferase reporter containing the wild-type 3′UTR of *FN1*. 293T cells were co-transfected with NC or miR-122 duplexes, pRL-TK and a firefly luciferase reporter plasmid carrying either the wild-type (WT) or the mutant (MUT) 3′UTR of *FN1*. ** *P* < .01; ns, no significant.

We further investigated how miR-122 attenuated expression of fibrosis-related genes. The above results revealed that overexpression of miR-122 downregulated FN1 expression at protein (Figure 2C and 2D; Supplementary Figure 3D) but not mRNA level (Supplementary Figures 2 and 3C). Consistently, antagonism of miR-122 significantly elevated the level of FN1 protein (Figure 3A). Furthermore, bioinformatic analysis using the RNAhybrid algorithm predicted a putative miR-122 binding site in the 3′-untranslated region (UTR) of *FN1* (Supplementary Figure 4). Dual-luciferase reporter analysis showed that co-expression of miR-122 significantly inhibited the activity of firefly luciferase with wild-type but not mutant 3′UTR of *FN1* (Figure 3B). These data indicate that miR-122 may directly suppress FN1 expression by binding to its 3′UTR.

**Figure 4:**
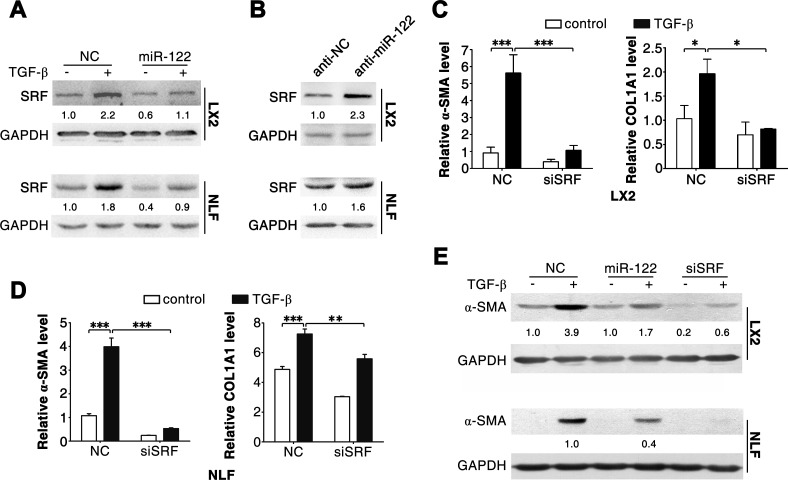
Silencing of SRF attenuates the effect of TGF-β in LX2 and NLFs (A) Transfection of miR-122 repressed the expression of SRF. (B) Inhibition of miR-122 elevated the level of SRF. (C and D) Knockdown of SRF attenuated the TGF-β-induced increase of α*-SMA* and *COL1A1* mRNA levels. (E) Inhibition of SRF impaired the TGF-β-induced expression of α-SMA protein. LX2 and NLFs transfected with the indicated duplex were stimulated with 2 ng/ml TGF-β (+) or remained untreated (−, control) for 48 hours before immunoblotting (A, B and E) or qPCR analysis (C and D). For (A-E), GAPDH was used as an internal control. For immunoblotting, the intensity of each band was densitometrically quantified. The levels of target genes in each sample were normalized by that of GAPDH. * *P* < .05; ** *P* < .01; *** *P* < .001.

We next explored the mechanisms responsible for miR-122-induced downregulation of α-SMA and COL1A1 expression. Serum response factor (SRF) is an identified target for miR-122 in HCC cells [6], and SRF interacts with myocardin-related transcription factor to drive transcription of α-SMA and COL1A1 [2, 13, 14]. As expected, we found that introduction of miR-122 repressed SRF expression (Figure 4A), whereas inhibition of endogenous miR-122 elevated SRF level (Figure 4B) in both LX2 and NLFs. Furthermore, similar to miR-122 overexpression, knockdown of SRF (Supplementary Figure 5) significantly attenuated TGF-β-induced elevation of α-SMA and COL1A1 levels (Figure 4C-E).

These results imply that miR-122 may suppress FN1 translation and attenuate SRF-induced transcription of α-SMA and COL1A1.

### Restoration of miR-122 expression inhibits hepatic fibrogenesis *in vivo*

To further evaluate the *in vivo* effect of miR-122 on hepatic fibrogenesis, a hepatic fibrosis model was first established by injecting mice with CCl_4_ twice a week for 4 weeks. The histopathological changes of the liver were visualized by hematoxylin and eosin (H&E) staining, and the collagen deposition was assessed by Sirius red staining. As reported, continuous CCl_4_ treatment resulted in hepatic necrosis and led to hepatic fibrosis (Supplementary Figure 6A). Furthermore, a significant downregulation of miR-122 was observed in the fibrotic livers collected from CCl_4_-treated mice, compared to the non-fibrotic livers isolated from vehicle-treated group (Supplementary Figure 6B). Consistently, miR-122 was also decreased in human cirrhotic livers compared to normal livers (Supplementary Figure 6C).

**Figure 5:**
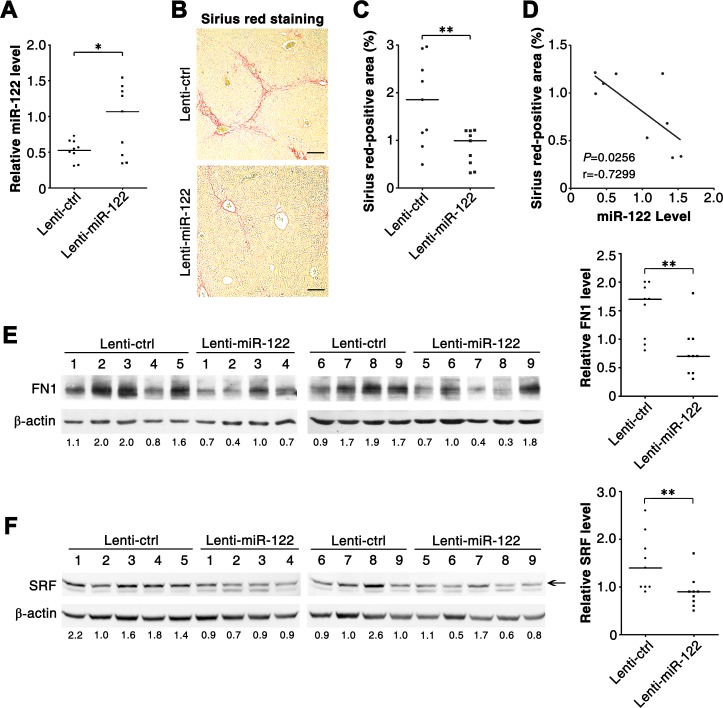
Reintroduction of miR-122 ameliorates hepatic fibrosis in CCl_4_-treated mice (A) Administration with miR-122-expressing lentiviruses enhanced miR-122 levels in murine livers. miR-122 level was analyzed by qPCR. Lenti-ctrl, negative control lentivirus without miRNA expression cassette; Lenti-miR-122, miR-122-expressing lentivirus. (B and C) Infection with Lenti-miR-122 inhibited collagen deposition *in vivo*. Collagen fibres in murine livers were stained in red by Sirius red staining. In (B), representative images are shown. In (C), the Sirius red-positive area relative to the total examined area was assessed to quantify the extent of fibrosis. (D) The extent of miR-122 elevation was significantly correlated with the extent of collagen reduction in the livers from Lenti-miR-122-injected mice. The correlation was analyzed by Spearman's correlation coefficient, using the data from (A) and (C). (E and F) Infection with Lenti-miR-122 inhibited the expression of FN1 and SRF *in vivo*. Protein extracts from liver tissues were subjected to immunoblotting for FN1 (E) and SRF (F). The intensity of each band was densitometrically quantified. The levels of target genes in each sample were normalized by that of β-actin (internal control). The median value of the normalized levels was set to 1, therefore the value under each sample indicates the relative level of target genes. The relative levels of target genes were subjected to statistical analysis using Mann-Whitney test. For (A-F), six-week-old Balb/c mice were treated with CCl_4_ twice a week for 4 weeks, and control (Lenti-ctrl) or miR-122-expressing lentiviruses (Lenti-miR-122) were administered intravenously at both the 5^th^ and 7^th^ days after the first CCl_4_-injection. Liver tissues were collected 48 hours after the last CCl_4_ treatment and subjected to qPCR analysis (A), Sirius red staining (B) and immunoblotting (E, F). Scale bar = 100 μm. * *P* < .05; ** *P* < .01.

We then explored whether reintroduction of miR-122 could inhibit CCl_4_-induced hepatic fibrosis *in vivo*. On both the 5^th^ and 7^th^ days after the first CCl_4_ injection, mice were administered intravenously with lentiviruses containing empty vector (control, Lenti-ctrl) or miR-122-expressing cassette (Lenti-miR-122). As shown, infection with Lenti-miR-122 significantly enhanced miR-122 level in murine livers (Figure 5A). Compared with Lenti-ctrl injection, treatment with Lenti-miR-122 markedly inhibited the formation of collagen fibrils in the livers of CCl_4_-treated mice, as shown by the decrease in Sirius red staining (Figure 5B and 5C). Significantly, the livers with more pronounced elevation of miR-122 displayed more obvious reduction of collagen fibrils (Figure 5D). Moreover, administration with Lenti-miR-122 obviously reduced the expression of FN1 and SRF protein in the livers of CCl_4_-treated mice (Figure 5E and 5F). These data suggest that miR-122 may suppress hepatic fibrogenesis *in vivo*.

We further showed that miR-122-induced remission of fibrosis was not attributed to the decrease of liver injury, because the serum alanine transaminase (ALT) levels were comparable between Lenti-ctrl- and Lenti-miR-122-treated mice (Supplementary Figure 7). To further dissect the cell population infected by lentiviruses, we detected the distribution of GFP, which was encoded by lentiviruses as a reporter. A significant colocalization of GFP with α-SMA was observed (Supplementary Figure 8), suggesting that fibrogenic cells are effectively infected by lentiviruses *in vivo*.

**Figure 6:**
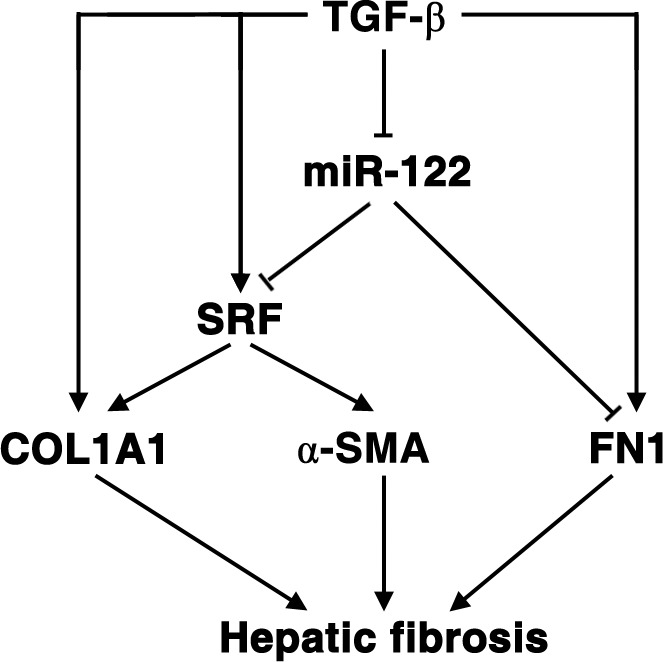
Schematic overview on the TGF-β-miR-122-FN1/SRF signaling cascade and its implication in hepatic fibrogenesis

Taken together, our results elucidate a novel TGF-β-miR-122-FN1/SRF signaling network and its implication in hepatic fibrogenesis (Figure 6).

## DISCUSSION

Most of the previous publications about miR-122 have been dedicated to exploring its biological function in hepatocytes, because miR-122 is present at approximately 50,000 copies per hepatocyte [15]. In this study, we detected a substantial miR-122 expression in HSCs and fibroblasts, and revealed an important role of miR-122 in repressing the activation of fibrogenic cells and the development of liver fibrosis.

TGF-β is one of the most potent fibrogenic cytokines in the liver [1]. We found that miR-122 level was significantly reduced in both human and mouse fibrotic livers. Furthermore, TGF-β treatment downregulated miR-122 expression, indicating that reduction of miR-122 level may represent a critical event to promote liver fibrosis. The mechanisms mediate miR-122 downregulation remains unclear. In classic pathway, TGF-β stimulation leads to activation of Smad2 and Smad3, which cooperates with Smad4 to transactivate the transcription of downstream genes. However, we could not identify any Smad binding element in the miR-122 promoter region, suggesting that TGF-β may regulate miR-122 expression indirectly and the underlying mechanisms need further investigation in the future.

During hepatic fibrogenesis, SRF is stimulated to drive the transcription of COL1A1 and α-SMA [2, 13, 14]. FN1 also increases in fibrotic liver. Type I collagen is the major component of fibril, fibronectin is required for the accumulation of ECM components, e.g. collagen type I [3], and α-SMA promotes contractile force and ECM stiffness [1, 2]. Furthermore, the interaction between FN1 with cell surface is essential for TGF-β-mediated activation of fibrogenic cells [16]. Here we showed that introduction of miR-122 inhibited the expression of FN1 and SRF, and inhibition of miR-122 elevated FN1 and SRF expression in both HSCs and fibroblasts. Consistently, the 3′-untranslated region (3′-UTR) of FN1 mRNA contained binding sequence of miR-122 and overexpression of miR-122 inhibited the activity of reporter with wild-type but not mutant 3′-UTR of FN1. Moreover, knockdown of SRF mimiced the suppressive effects of miR-122 on TGF-β-induced increase of α-SMA and COL1A1. More importantly, introduction of miR-122 into CCl_4_-treated mice attenuated the expression of FN1 and SRF in fibrotic livers, and alleviate hepatic fibrosis *in vivo*. All these data suggest that FN1 and SRF are bona fide targets of miR-122, and miR-122 may suppress hepatic fibrosis by simultaneously blocking multiple aspects of fibrogenesis, including collagen production, fibril assembly and ECM contraction. In agreement with our findings, it is recently reported that miR-122 is downregulated in HSCs from CCl_4_- and bile duct ligation-induced fibrotic livers of mice and miR-122 inhibits collagen maturation *in vitro* by targeting P4HA1, a component of prolyl 4-hydroxylase that promotes the procollagen to form triple helix of collagen molecule [17]. There are two general types of chronic liver injuries, hepatocellular and cholestatic injury, may result in hepatic fibrosis [18]. HSCs are the major source of activated fibrogenic cells in hepatocellular injury and fibroblasts play a critical role in the pathogenesis of cholestatic liver fibrosis [19]. Taken together, miR-122 may repress different etiologies-elicited hepatic fibrosis by suppressing activation of both HSCs and fibroblasts.

Emerging evidence suggests that aberrant expression of miRNAs may contribute to hepatic fibrosis via different mechanisms. For example, miR-29b [20] and miR-133a [21] directly represses the expression of several collagen genes, whereas miR-19b [22], miR-101 [23] and miR-146a [24] impairs TGF-β signaling. miR-221/222 promotes proliferation [25], and miR-15b/16 induces apoptosis in activated HSCs [26]. To date, most of the studies have demonstrated the anti-fibrosis effects of miRNAs *in vitro* but hardly *in vivo*. Herein, we revealed that infection with miR-122-expressing lentiviruses significantly enhanced miR-122 levels and meanwhile reduced the amount of FN1 and collagen fibrils in the livers of CCl_4_-treated mice. Furthermore, the extent of miR-122 elevation was significantly correlated with that of collagen reduction. These results provide *in vivo* evidences to support the anti-fibrosis role of miR-122. It is known that efficient lentiviral transduction *in vivo* requires cell cycling [27]. At the early stage of CCl_4_ treatment, the hepatocytes undergo necrosis, whereas the fibrogenic cells are stimulated to proliferate. Consistently, lentiviruses, which were systemically administered on the 5th and 7th day after the first CCl_4_ injection, mainly infected the fibrogenic cells. Furthermore, Lenti-miR-122-treated mice displayed similar level of liver function enzyme ALT, compared with Lenti-ctrl-injected group. These findings imply that the remission of liver fibrosis in our model may be attributed to the suppressive effects of miR-122 on fibrogenic cells but not the decrease of hepatocyte injury.

It is well known that liver fibrosis is a risk factor for HCC [28]. Previous studies from us and others have demonstrated the inhibitory function of miR-122 on proliferation, metastasis and angiogenesis of HCC [6-10]. Here we found that TGF-β significantly downregulated miR-122 expression, whereas restoration of miR-122 expression dramatically attenuated fibrosis-promoting effect of TGF-β by directly suppressing the expression of FN1 and SRF, and subsequently abrogating the transcription of COL1A1 and α-SMA in HSCs and fibroblasts. This study discloses a novel TGF-β-miR-122-FN1/SRF signaling cascade and further identifies the importance of miR-122 in preventing hepatic fibrogenesis and therefore substantially extends our understanding about the function of miR-122 and the molecular mechanisms of hepatic fibrosis.

## MATERIALS AND METHODS

The reagents and experimental details are described in the Supplementary Materials and Methods.

### Primary cells and cell lines

The cells used in this study include primary hepatocytes and HSCs isolated from mouse livers, primary fibroblasts isolated from human normal liver (NLFs) or human skin (SFs), an immortalized human HSC cell line LX2 (LX2 was kindly provided by Dr. S. L. Friedman, Mount Sinai School of Medicine, New York), and the transformed human embryonic kidney cell line HEK293T.

To isolate mouse primary hepatocytes and HSCs, adult male mice at ages of 8-10 weeks were anesthetized and subjected to two-step liver perfusion sequentially with ethylene glycol bis (2-aminoethyl) tetraacetic acid (EGTA) and collagenase (Sigma, St. Louis, MO, USA) via the portal vein. To isolate hepatocytes, the disaggregated liver tissues were minced, filtered through a 100-μm cell strainer and centrifuged at 50 *g* for 2 min to collect the cell pellets, which were then resuspended in D-Hank's solution, mixed with the same volume of PERCOLL (GE Healthcare, Waukesha, WI, USA) and centrifuged at 50 *g* for 15 min to collect the pellets of viable hepatocytes. To isolate HSCs, the disaggregated liver tissues were minced and further digested in D-Hank's solution containing DNase I, followed by filtering through a 100-μm cell strainer and centrifugation at 50 *g* for 2 min. The hepatocyte pellets were discarded. The supernatants were further centrifuged at 550 *g* for 6 min to collect the cell pellets, which were resuspended in DMEM and further purified by single-step density gradient centrifugation with 11.5% (w/v) Iodixanol (OptiPrep™, Axis-Shield Point-of-Care Division, Oslo, Norway). HSCs were collected by aspirating of the white top layer of the gradient.

NLFs were extracted from normal liver tissues of patients who underwent resection of hepatic hemangiomas. Liver tissues were minced and digested at 37°C with agitation for 6 hours in RPMI 1640 medium supplemented with 10% FBS, collagenase and hyaluronidase (Sigma). The dissociated tissues were kept still at 37°C for 5 min to allow the sedimentation of tissue debris and hepatocytes. The stromal cells that remained in the supernatant were collected by centrifugation at 250 *g* for 5 min and were then grown in PRMI 1640 medium supplemented with 10% FBS. Fibroblasts were selected and enriched due to their growth advantage, and fibroblasts at passages 5-10 were used.

To isolate SFs, fat and loose fascia was trimmed from foreskin tissues. The tissues were then cut into strips that were approximately 1 cm^2^ in area and were digested with trypsin at 4°C for 12 hours. The dermis was separated from the epidermis and further digested with collagenase at 37°C for 30 min, followed by filtering through a 100-μm cell strainer and centrifugation at 1000 *g* for 5 min to collect the fibroblasts.

Primary hepatocytes, HSCs, NLFs and SFs were grown in RPMI 1640 medium supplemented with 10% FBS, 100 U/mL penicillin and 100 μg/mL streptomycin. LX2 and HEK293T cells were cultured in DMEM with 10% FBS.

### Oligonucleotides and plasmids

We used the following miRNA and small interfering RNA (siRNA) oligonucleotides (Genepharma, Shanghai, China): miR-122 mimics, and siSRF targeting human *SRF* (901–921 nt, NM_003131.2) transcript. The negative control RNA duplex (NC) for both miRNA and siRNA was non-homologous to any human genome sequence. The miR-122 inhibitor (anti-miR-122), which was complementary to the sequence of mature miR-122, and its control (anti-NC) consisted of 2′-O-methyl-modified oligonucleotides (RiboBio, Guangzhou, China). All oligonucleotide sequences are listed in Supplementary Table 1.

Firefly luciferase reporter plasmid was used to verify the miR-122-targeted 3′ untranslated region (UTR). Lentivirus expression vector pCDH-miR-122 was utilized to express the mouse miR-122 precursor (mmu-pre-miR-122).

### Cell transfection and infection

RNA oligonucleotides were transfected into HSCs, NLFs and SFs using Lipofectamine 2000 (Invitrogen, Carlsbad, CA, USA) and into LX2 cells with Lipofectamine RNAiMAX (Invitrogen). A final concentration of 50 nM RNA duplex or 100 nM miRNA inhibitor was used, unless otherwise indicated. Lipofectamine 2000 was also used to transfect a mixture of plasmid/RNA oligonucleotides. For *in vivo* infection, mice were injected intravenously with lentiviruses (3×10^8^ vg in 100 μl 1×PBS).

### Cell contraction assay

Three dimensional collagen matrix (1 mg/ml) containing LX2 cells (3×10^5^ cells/ml) was incubated for 1 hour at 37 ºC to allow the formation of collagen lattice. The collagen lattice was further incubated for 24 hours in 10% FBS-containing DMEM with or without TGF-β, and then released from the tissue culture well. Twenty-four hours later, the collagen lattice was stained with crystal violet and the contraction of collagen gel was measured.

### Analysis of gene expression

Semiquantitative RT-PCR and real-time quantitative RT-PCR (qPCR) were used to detect RNA levels. Immunobloting was performed to evaluate protein levels.

### Animal studies

To induce hepatic fibrosis, BALB/c male mice were intraperitoneally injected with CCl_4_ (0.6 ml/kg body weight, mixed with corn oil at 1:4) twice a week for 4 weeks. Age-matched mice were treated with corn oil only as vehicle control. All experiments using mice were conducted in accordance with the Guide for the Care and Use of Laboratory Animals (NIH publications No. 80–23, revised 1996) and according to the institutional ethical guidelines for animal experiments.

### Human tissues

Normal liver tissues were collected from patients who underwent resection of hepatic hemangiomas. Fourteen fibrotic livers with chronic HBV (n = 12) or HCV (n = 1) infection or alcohol abuse (n = 1) were collected at the Third Affiliated Hospital and Cancer Center at Sun Yat-sen University. All of the tissues were histologically confirmed. Informed consent was obtained from each patient, and the study was approved by the Institute Research Ethics Committee.

### Fibrosis assessment

Formalin-fixed, paraffin-embedded mouse livers were cut into 5-μm sections, placed on polylysine-coated slides, deparaffinized in xylene and rehydrated through graded ethanol. Sections were stained with picrosirius red (0.1% Direct Red 80 in saturated picric acid) for 60 minutes, washed in 0.5% acetic acid, dehydrated in ethanol, cleared in xylene, and mounted in non-aqueous mounting medium. Ten random non-overlapping fields (magnification 100×) were acquired for each specimen. The collagen-staining area was digitized using Image-Pro^®^ Plus software (version 6.0, Media Cybernetics, Bethesda, MD, USA), which uses color cube-based selection criteria to ensure that only stained regions are counted. The stained area relative to the total examined area was assessed to quantify the extent of fibrosis.

### Statistical analysis

The data are presented as the mean ± standard error mean (SEM) from at least three independent experiments. The differences between groups were analyzed using Student's *t* test when only two groups were compared. Two-factor analysis was performed using two-way ANOVA with a post test for subsequent comparisons of individual factors. Analyses were performed using the GraphPad Prism program (version 4.0, GraphPad Software, Inc., San Diego, CA, USA). All statistical tests were two-sided and *P* < 0.05 was considered to be statistically significant.

## SUPPLEMENTARY MATERIALS AND METHODS FIGURES AND TABLES


