# Supplementary material for: Identification of a novel TGF-β-miR-122-fibronectin 1/serum response factor signaling cascade and its implication in hepatic fibrogenesis
Source: Oncotarget. 2015 Mar 26;6(14):12224–33. doi: 10.18632/oncotarget.3652 (PMC4494934; doi:10.18632/oncotarget.3652)
Supplement: Supplementary file 1 [file oncotarget-06-12224-s001.pdf]

Identification of a novel TGF-β-miR-122-fibronectin 1/serum response factor signaling cascade and its implication in hepatic fibrogenesis

SUPPLEMENTARY MATERIALS AND METHODS

Reagents

Reagents were purchased as follows: CCl_4 (Merck, Darmstadt, Germany); corn oil, Direct Red 80 and type I collagen from rat tail (Sigma, St. Louis, MO, USA); recombinant human TGF-β (R&D Systems, Minneapolis, MN, USA); TRIzol reagent (Invitrogen, Carlsbad, CA, USA); Moloney murine leukemia virus (MMLV) reverse transcriptase (Promega, Madison, WI, USA); restriction enzymes (MBI Fermentas, Hanover, MD, USA); Dulbecco's modified Eagle's medium (DMEM) and Roswell Park Memorial Institute (RPMI) 1640 medium (Hyclone, Logan, UT, USA); fetal bovine serum (FBS, GIBCO, Grand Island, NY, USA).

The following antibodies were used for immunoblotting: mouse monoclonal antibody against FN1 (610077, BD Biosciences, San Jose, CA, USA), α-tubulin (BM1452), β-actin (BM0627) or GAPDH (BM1623, Boster, Wuhan, China); rabbit polyclonal antibodies against α-SMA (ab5694, Abcam, Cambridge, MA, USA), SRF (AB60758a, BBI, Shanghai, China); ImmunoPure Peroxidase Conjugated Goat Anti-Mouse IgG (H+L) (cat. 31430) or Anti-Rabbit IgG (H+L) (cat. 31460, Thermo Scientific, Waltham, MA, USA).

Analysis of gene expression

For semiquantitative RT-PCR and real-time quantitative RT-PCR (qPCR)

analyses, total RNA from cells was extracted using the TRIzol reagent, subjected to DNase I digestion (1U/μL, Fermentas) at 37°C for 30 min and then to heat inactivation in DNase I at 65°C for 10 min.

The expression levels of miR-122 and the reference gene U6 were quantified using the TaqMan MicroRNA Assay kit (Applied Biosystems, Foster City, CA, USA). qPCR analyses for the expression of pri-miR-122, α-SMA, COL1A1, FN1 and the reference gene GAPDH were performed using Power SYBR® Green PCR Master Mix (Applied Biosystems). The temperature cycle profile for the qPCR reactions was 95 °C for 1 min and 40 cycles of 95 °C for 15 s and 60 °C for 1 min.

When Power SYBR® Green PCR Master Mix was used, melting curve analysis was performed to verify the specificity of the PCR product immediately after amplification, as follows: heating to 95 °C for 20 s, cooling to 60 °C for 20 s, followed by a temperature increase to 95°C with a transition rate of 0.11°C /s and the continuous detection of fluorescence.

All qPCR reactions were performed on a LightCycler 480 (Roche Diagnostics, Germany), and were run in triplicate. The cycle threshold (Ct) values did not differ by more than 0.5 among the triplicate runs. The level of target genes was normalized to the levels of the internal control genes to yield a $2^{-\triangle\triangle Ct}$ value. Sequences for primers are listed in Supplementary Table 1.

Plasmids

The details for plasmid construction are described below. The lentivirus vector

pCDH-miR-122 was generated by cloning the genomic fragment (621 bp) that encompasses the mouse miR-122 precursor and its 5'- and 3'-flanking sequences into the *Xba*I and *Bam*HI sites of pCDH-CMV-MCS-EF1-copGFP (System Biosciences, Mountain View, CA, USA).

To verify the miR-122-targeted 3'UTR, a pGL3cm-FN1-3'UTR-WT was created by inserting the full length 3'UTR (1090 bp) of human *FN1* into the *Eco*RI and *Xba*I sites downstream of the stop codon of firefly luciferase in pGL3cm [1], which was previously produced based on the pGL3-control (Promega). The pGL3cm-FN1-3'UTR-MUT plasmid, which carried the mutated sequence in the complementary site for the seed region of miR-122, was generated by fusion PCR based on the pGL3cm-FN1-3'UTR-WT vector.

All constructs were confirmed by direct sequencing, and all primer sequences used for cloning are listed in Supplementary Table 1.

Luciferase reporter assay

To verify the miR-122-targeted 3'UTR, 293T cells grown in a 48-well plate were co-transfected with 10 ng of firefly luciferase reporter, 20 ng of pRL-TK (Promega) and 20 nM of NC or miR-122 duplex. pRL-TK, which expresses *Renilla* luciferase, was used to correct the differences in both transfection and harvest efficiencies. Forty-eight hours post-transfection, cells were harvested and subjected to the luciferase assay as previously described [1].

Virus production

For lentivirus production, HEK293T cells were co-transfected with the lentivirus expression vector and the Lenti-X™ HTX Packaging System (Clontech, Takara Bio Inc., Dalian, China), followed by replacement with fresh medium 16 hours post-transfection and the supernatant was harvested 48 hours later. The lentiviral supernatant was centrifuged briefly (1000 *g* for 10 min) to remove cellular debris and the aliquots were stored at –80 °C. For *in vivo* infection, the lentiviral supernatant was further concentrated using Lenti-X™ Concentrator (Clontech, Takara Bio Inc.). The virus pellet was resuspended in 1×PBS and titrated by qPCR analysis.

Analysis of transaminase activity

Serum samples were collected from mice and stored at -80°C. The alanine transaminase (ALT) was tested using the serum transaminase test kit (Jiancheng, Nanjing, China) following the supplier's instruction.

Immunohistochemical staining (IHC)

Formalin-fixed, paraffin-embedded tissues were cut into 5 μm sections, placed on polylysine-coated slides, deparaffinized in xylene, rehydrated through graded ethanol, quenched for endogenous peroxidase activity in 0.3% hydrogen peroxide, and processed for antigen retrieval by pressure cooking in 10 mM citrate buffer (pH 6.0). Sections were incubated at 4 °C overnight with anti-α-SMA (ZM-0003, ZSGB-BIO, Beijing, China) and anti-TurboGFP(d) antibody (AB513, Evrogen JSC, Moscow, Russia) diluted 1:200 and 1:5000 respectively in Antibody Diluent with Background Reducing Components (DakoCytomation, Glostrup, Denmark). Immunostaining was performed using ChemMate DAKO EnVision Detection Kit, Peroxidase/DAB,

Rabbit/Mouse (code K5007, DakoCytomation), which resulted in a brown-colored precipitate at the antigen site. Subsequently, sections were counterstained with hematoxylin (Zymed Laboratories, South San Francisco, CA) and mounted in non-aqueous mounting medium.

Bioinformatic tools

RNAhybrid algorithm (http://bibiserv.techfak.uni-bielefeld.de/rnahybrid/) was used to predict the pupative miRNA binding sites.

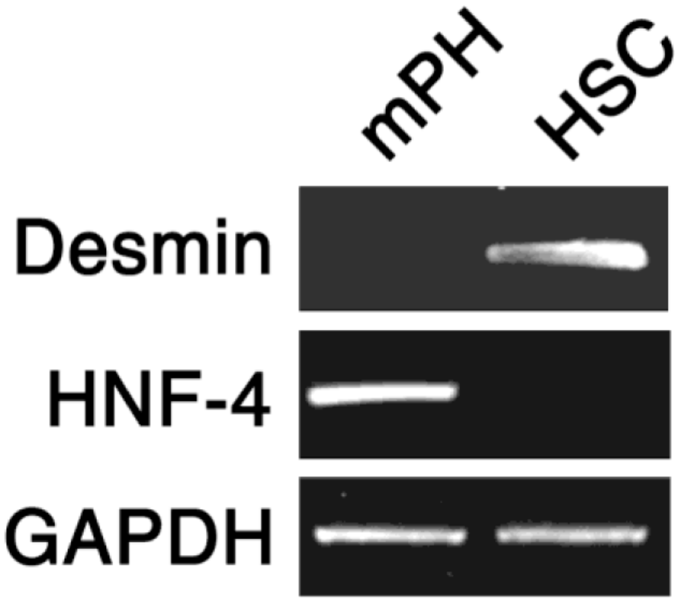

Supplementary Figure 1: Cell-specific markers in murine primary hepatocytes and HSCs. Primary hepatocytes (mPH) and HSCs isolated from mice were analyzed for *Desmin* (marker for HSCs) and *HNF-4* (marker for hepatocytes) by RT-PCR analysis. *GAPDH* was used as an internal control.

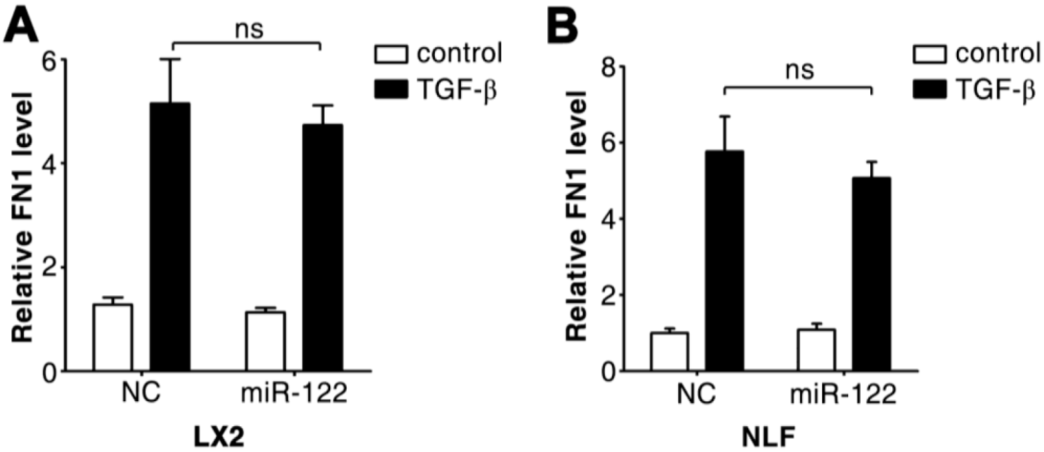

Supplementary Figure 2: Restoration of miR-122 expression has no effect on the mRNA level of *FN1*. LX2 (A) and NLF (B) cells were transfected with negative control (NC) or miR-122 duplex for 24 hours, and then stimulated with 2 ng/ml TGF-β or remained untreated (control) for 48 hours before qPCR analysis. The level of *FN1* was normalized to that of *GAPDH*. ns, no significant.

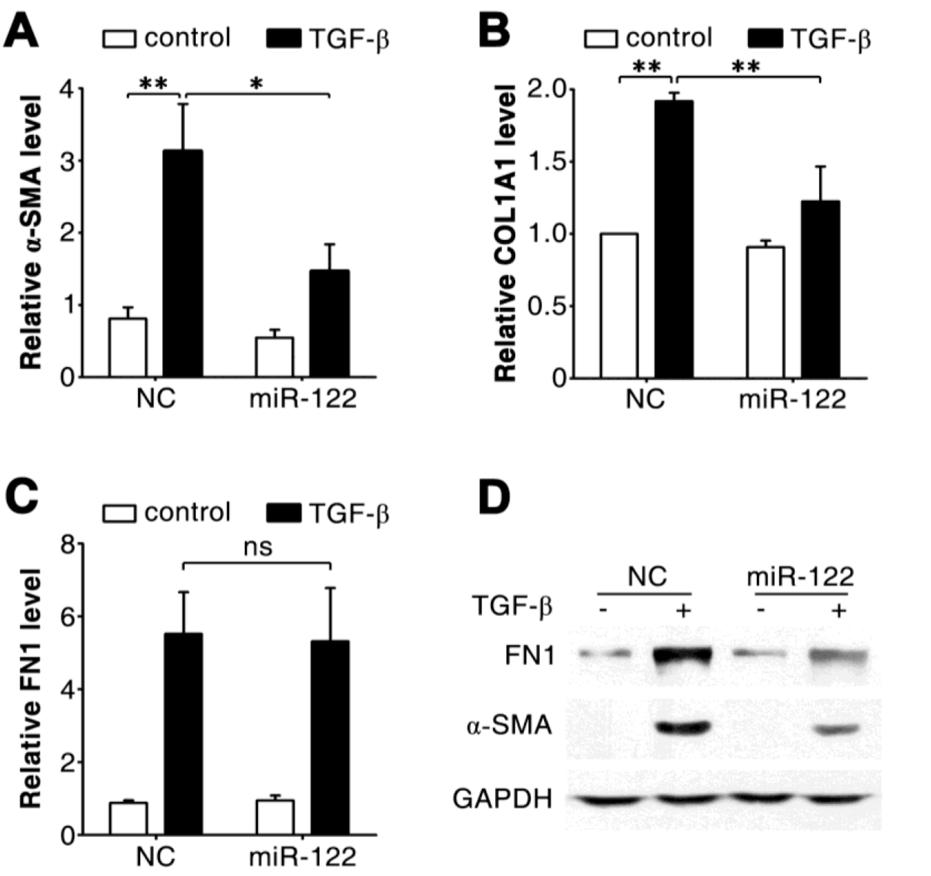

Supplementary Figure 3: Introduction of miR-122 inhibits the TGF-β-induced expression of fibrosis-related genes in human skin fibroblasts. Human skin fibroblasts (SFs) transfected with negative control (NC) or miR-122 duplex were stimulated with 2 ng/ml TGF-β (+) or remained untreated (control, -) for 48 hours before qPCR analysis for *α-SMA* (A), *COL1A1* (B) and *FN1* (C) or immunoblotting (D). GAPDH were used as an internal control for immunoblotting. For qPCR analysis, the levels of target genes were normalized to the expression of *GAPDH*. * *P* < .05; ** *P* < .01; ns, no significant.

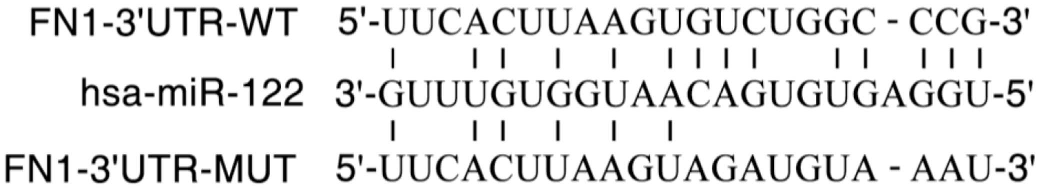

Supplementary Figure 4: miR-122 and its putative binding sequences in the 3'UTR of *FN1*. Wild-type miR-122 sequence and the wild-type and mutant 3'UTR segment of *FN1* are shown. Mutations were generated in the complementary site that binds to the seed region of miR-122.

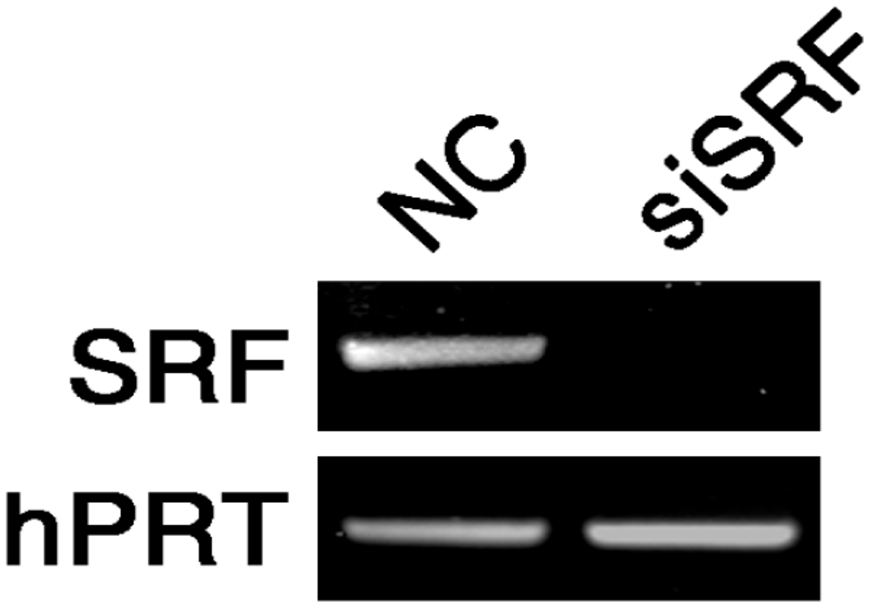

Supplementary Figure 5: siRNA silences the endogenous SRF expression. NLF cells were transfected with the indicated duplex for 48 hours and then subjected to RT-PCR analysis. *hPRT* was used as an internal control.

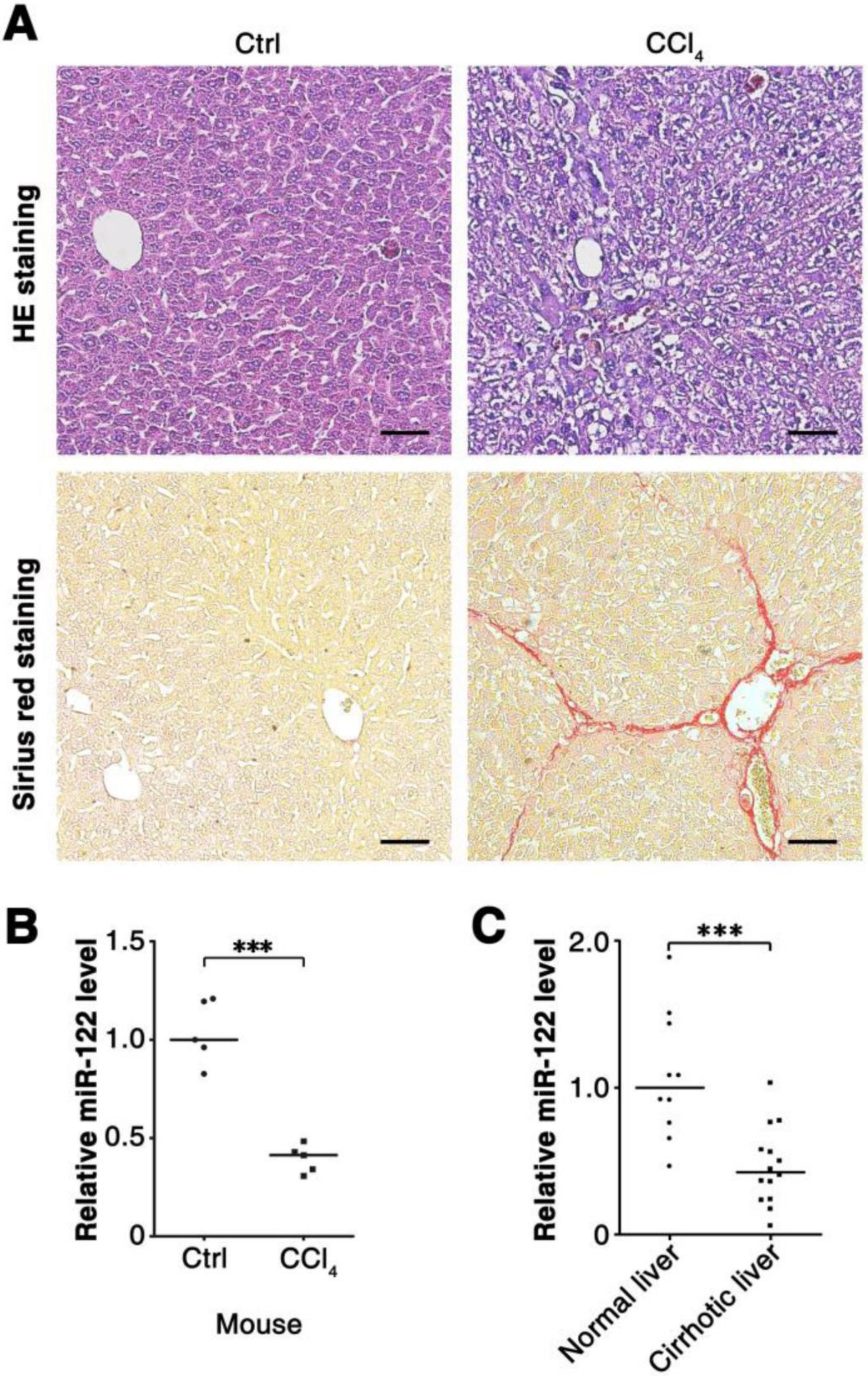

Supplementary Figure 6: miR-122 is downregulated in fibrotic livers. (A) Induction of hepatic fibrosis in mice. Mice were treated with CCl_4 for 4 weeks, and

fibrosis was assessed by H&E (top) and Sirius red (bottom) staining. Scale bars = 100 µm. (B) miR-122 was downregulated in mouse fibrotic livers. Balb/c mice were treated with CCl_4 or corn oil (vehicle control) for 4 weeks. miR-122 expression in livers from vehicle- (Ctrl) and CCl_4-treated mice was analyzed by qPCR. (C) miR-122 expression was reduced in human cirrhotic livers. miR-122 levels were analyzed in human normal livers and cirrhotic livers. *** $P < .001$.

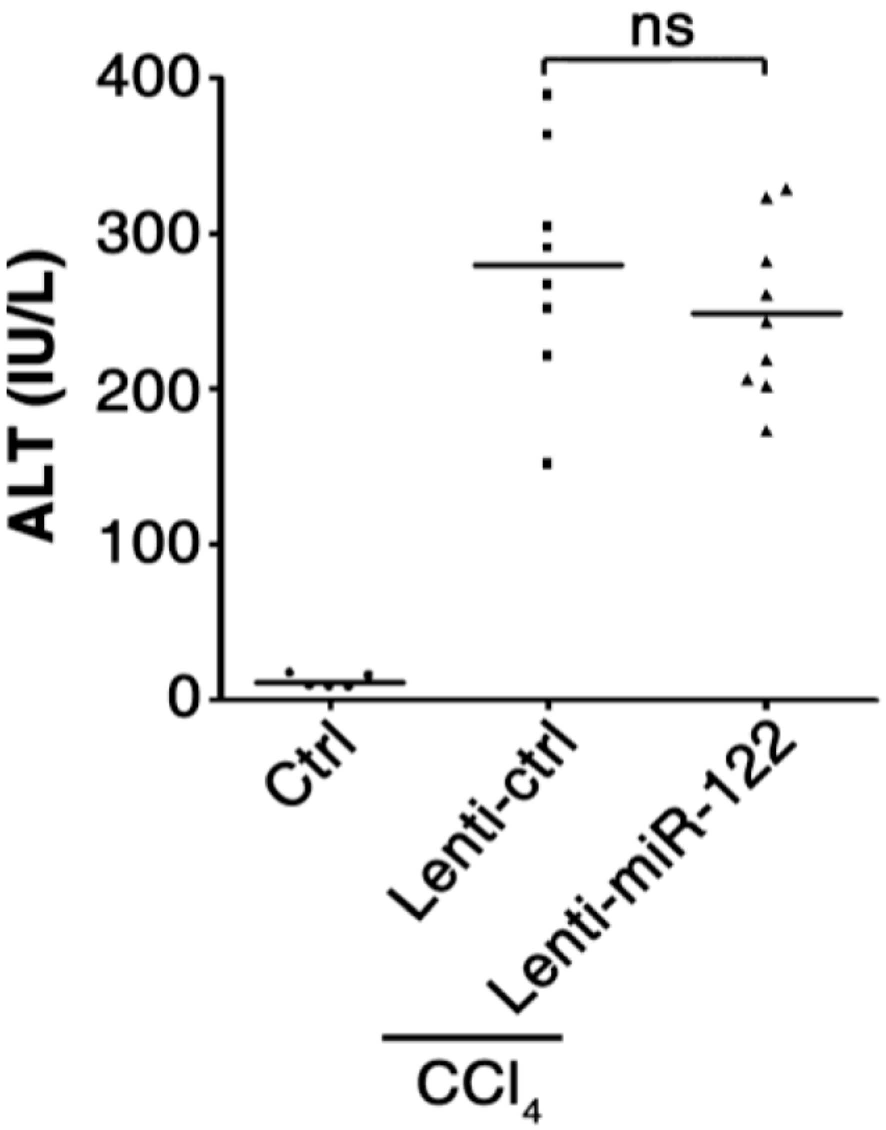

Supplementary Figure 7: Analysis of alanine transaminase activity in lentivirus-administered mice. Alanine transaminase (ALT) activity was detected in the sera from non-treated (Ctrl) and CCl_4-treated mice that were injected with control (Lenti-ctrl) or miR-122-expressing lentiviruses (Lenti-miR-122, as in Figure 5). ns, no significant.

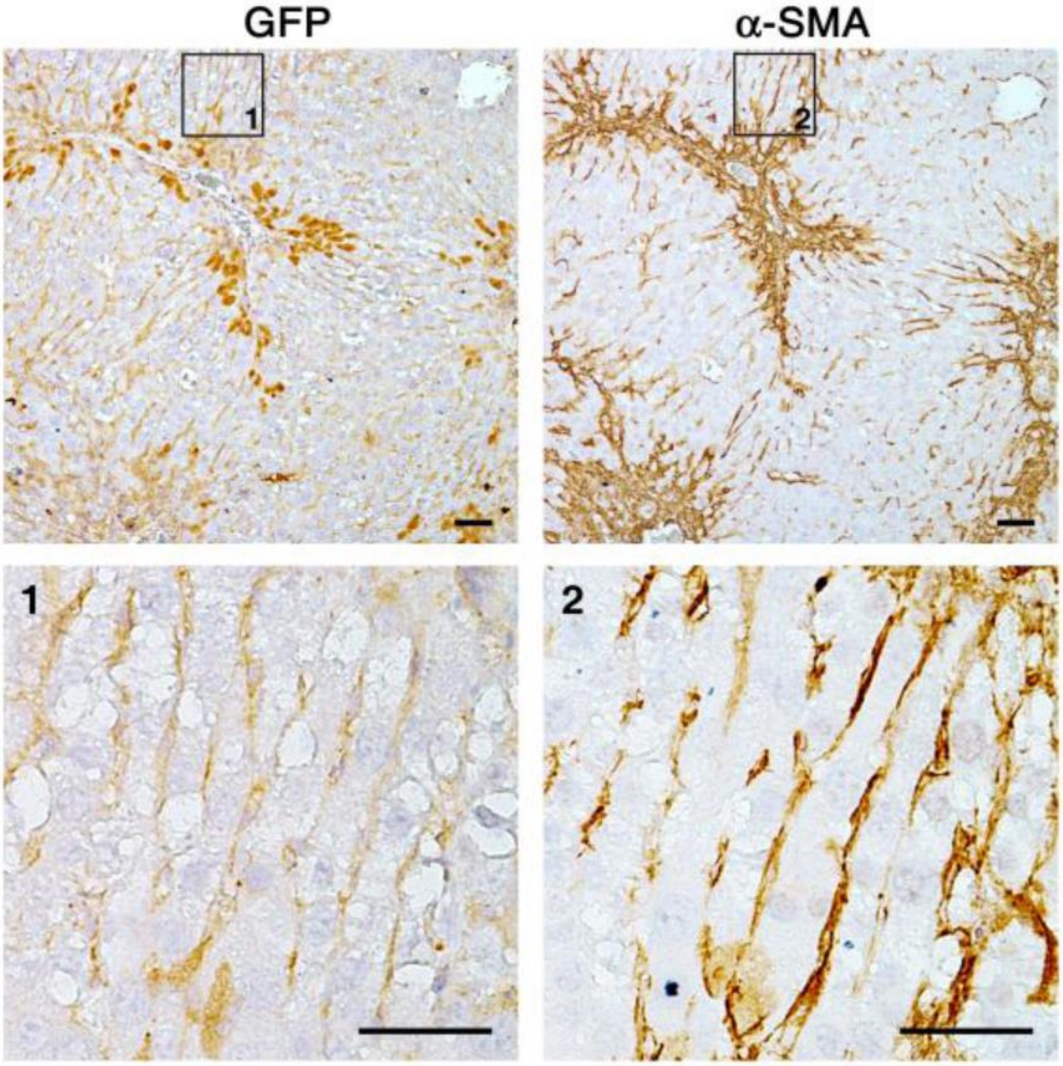

Supplementary Figure 8: Fibrogenic cells in the liver are infected by lentiviruses. Liver tissues were collected from CCl_4-treated mice that were injected with lentiviruses (as in Figure 5). Lentivirus-expressing GFP and fibrogenic cell-expressing α-SMA were detected in serial sections by immunohistochemical staining. Scale bar = 100 μm.

Supplementary Table 1. Sequences of RNA and DNA Oligonucleotides

Name	Sense Strand/Sense Primer (5'-3')	Antisense Strand/Antisense Primer (5'-3')
**miRNA and siRNA duplexes**		
miR-122	UGGAGUGUGACAAUGGUGUUUG	AACACCAUUGUCACACUCAUUU
NC	UUGUACUACACAAAAGUACUG	GUACUUUUGUGUAGUACAGUU
siSRF	GCGUGAAGAUCAAGAUGGAdTdT	UCCAUCUUGAUCUUCACGCdGdG
anti-NC	GUGGAUAUUGUUGCCAUCA	
anti-miR-122	CAAACACCAUUGUCACACUCCA	
**Primers for qRCR or RT-PCR**		
α-SMA	ACTGGGACGACATGGAAAAG	GCGTCCAGAGGCATAGAGAG
COL1A1	AGCCAGCAGATCGAGAACAT	CAGTTCTTCTGGGCCACACT
FN1	CAGTGGGAGACCTCGAGAAG	TCCCTCGGAACATCAGAAAC
SRF	GCCACATGATGTACCCTAGC	ACTGTCCCAGATGATGCTGT
Desmin (mouse)	AGGAGAGCAGGATCAACCTT	CCTGGCTTACAGCACTTCAT
HNF-4 (mouse)	CTGAAGGTGCCAACCTCAAT	CACATTGTCGGCTAAACCTG
GAPDH (human)	GAGTCAACGGATTTGGTCGT	GACAAGCTTCCCGTTCTCAG
GAPDH (mouse)	AACTTTGGCATTGTGGAAGG	CACATTGGGGGTAGGAACAC
**Primers for cloning (Restriction enzyme sites were underlined)**		
mmu-miR-122	ATGTCTAGAAACCATGACGAGGTGAGAG	ATGGGATCCTGGGTGTCAGGGTAGTCAGT
FN1-3'UTR	ATGGAATTCAATCATCTTTCCAATCCAGA	CGCTCTAGAAGTCATTTTTATTTCCCTTG

SUPPLEMENTARY REFERENCE

1. Su H, Yang JR, Xu T, Huang J, Xu L, Yuan Y, Zhuang SM. MicroRNA-101, down-regulated in hepatocellular carcinoma, promotes apoptosis and suppresses tumorigenicity. Cancer Res. 2009; 69:1135-1142.
